# Perceived differences on the role of traditional birth attendants in rural Tanzania: a qualitative study

**DOI:** 10.1186/s12884-021-03611-0

**Published:** 2021-02-15

**Authors:** Yoko Shimpuku, Frida E. Madeni, Kana Shimoda, Satoe Miura, Beatrice Mwilike

**Affiliations:** 1 Graduate School of Biomedical and Health Sciences, Hiroshima University, 1-2-3 Kasumi, Minami-ku, Hiroshima, 734-8551 Japan; 2 Magunga District Hospital, P. O. Box 430, Old-Korogwe, Tanga, Tanzania; 3 Graduate School of Nursing Science, St. Luke’s International University, 10-1 Akashi-cho, Chuo-ku, Tokyo, 104-0044 Japan; 4 Kyoto University Hospital, 53 Shogoin-kawaharacho, Sakyo-ku, Kyoto, 606-8507 Japan; 5 School of Nursing, Muhimbili University of Health and Allied Sciences, P. O. Box 65001, Dar es Salaam, Tanzania

**Keywords:** Childbirth, Culture, Traditional birth attendants, Local knowledge, Tanzania, Africa

## Abstract

**Background:**

In many low to middle income countries, traditional birth attendants (TBAs) play various roles (e.g., provision of health education, referral to hospitals, and delivery support) that can potentially improve women’s access to healthcare. In Tanzania, however, the formal healthcare systems have not acknowleded the role of the TBAs. TBAs’ contributions are limited and are not well described in policy documents. This study aimed to examine the perspectives of both TBAs and skilled birth attendants (SBAs) to clarify the role of TBAs and issues impacting their inclusion in rural Tanzania.

**Methods:**

We used a qualitative descriptive design with triangulation of investigators, methods, and data sources. We conducted semi-structured interviews with 15 TBAs and focus group discussions with 21 SBAs in Kiswahili language to ask about TBAs’ activities and needs. The data obtained were recorded, transcribed, and translated into English. Two researchers conducted the content analysis.

**Results:**

Content analysis of data from both groups revealed TBAs’ three primary roles: emergency delivery assistance, health education for the community, and referrals. Both TBAs and SBAs mentioned that one strength that the TBAs had was that they supported women based on the development of a close relationship with them. TBAs mentioned that, while they do not receive substantial remuneration, they experience joy/happiness in their role. SBAs indicated that TBAs sometimes did not refer women to the hospital for their own benefit. TBAs explained that the work issues they faced were mainly due to insufficient resources and unfavorable relationships with hospitals. SBAs were concerned that TBAs’ lacked formal medical training and their actions could interfere with SBAs’ professional work. Although there were no between-group interactions at the time of this study, both groups expressed willingness to collaborate/communicate to ensure the health and lives of mothers and babies.

**Conclusions:**

TBAs and SBAs have different perceptions of TBAs’ knowledge and skills, but agreed that TBAs need further training/inclusion. Such collaboration could help build trust, improve positive birth experiences of mothers in rural Tanzania, and promote nationwide universal access to maternal healthcare.

**Supplementary Information:**

The online version contains supplementary material available at 10.1186/s12884-021-03611-0.

## Background

Since adopting the Sustainable Development Goals (adopted by the UN member states in 2015), Sub-Saharan African countries have found it challenging to achieve the related maternal health promotion targets. The global aim is to limit the maternal mortality  ratio (MMR) to 70/100,000 live births [1]. In rural areas of Tanzania, nearly half the women still give birth outside health facilities, so these areas continue to experience a high MMR and raise the national MMR of 556/100,000 live births [2]. Despite fertility rates being higher in rural areas of Tanzania, health workers and facilities remain concentrated in urban areas. Hence, homebirth rates are much higher in rural parts of the country than in urban areas (urban: 16.8% vs. rural: 56.1%) [2].

The healthcare system of Tanzania faces difficulties in functioning properly owing to several factors, including increasing population, shortage of healthcare providers and medical materials and facilities, decreasing motivation of healthcare providers, and the double health burdens of communicable and non-communicable diseases [3]. Although the government has been aiming to increase the number of facilities, the development of human resources in the healthcare field remains a big challenge in the country. The health workforce of Tanzania relies heavily on practitioners who have received brief training courses, with nurses and midwives accounting for 27% of the total national healthcare workforce. This percentage is much lower than that of other African countries (about 50%) [3].

Regarding strategies to promote universal access to pregnancy and delivery care, the incorporation of traditional birth attendants (TBAs) into the healthcare system alongside formal healthcare providers (i.e., skilled birth attendants [SBAs]) has generally been shown to be an acceptable strategy in the context of low to middle income countries [4–6]. Using the World Health Organization’s definition [7], a TBA is, “a person who assists the mother during childbirth, and who initially acquired her skills by delivering babies or through an apprenticeship with other TBAs.” The United Nations Population Fund evaluated TBA trainings in 1996. They concluded that, in settings where women-prefer TBAs—such as areas with an insufficient number of SBAs and limited access to healthcare facilities—TBA training may be the only means to optimize the use of community-level heath workers to promote maternal child health. The trainings may also be necessary to improve the quality of care provided by TBAs in such cases [8]. In a more recent Cochrane review, TBA training was found to be effective in improving outcomes—lowering perinatal death, stillbirth, and neonatal death rates—for cases attended by TBAs. Maternal death rates were also lower, but the differences were not statistically significant compared to cases attended by untrained TBAs [6].

However, as TBAs are segregated from the formal healthcare system in Tanzania, their potential contributions are limited and not clearly described in the national policy [9]. This view was corroborated by a study that reported poor linkage between TBAs and the formal health system in Tanzania [10]. Although research related to the activities of TBAs is limited, we sought out some reports from other countries about the inclusion of TBAs in their health systems. In Kenya [11] and Somaliland [12], TBAs receive incentive payments for referrals. In Sierra Leone, Somaliland [13] and China [14], they are regarded as connectors between the community and healthcare services. In Bangladesh, they have been reported as breastfeeding supporters [15]. In such studies, researchers have repeatedly described TBAs as trusted members of the community and remarked on their value for increasing women’s access to national healthcare systems. The relevance of their work in this specific context highlights the need to examine the ongoing activities of TBAs and identify the issues limiting their inclusion in Tanzania’s formal healthcare system. Hence, this study aimed to describe the perceptions of TBAs and SBAs regarding TBAs’ roles and the issues surrounding their inclusion in the formal healthcare system in rural Tanzania.

## Methods

### Design

We utilized a descriptive cross-sectional design with a qualitative approach. Data were collected through individual interviews with TBAs and focus group discussions with SBAs working in a district hospital in rural Tanzania. To ensure a comprehensive understanding of the studied phenomena, triangulation is recommended for qualitative studies [16]; therefore, we used triangulation of investigation, methods, and data sources. Investigators included both Japanese and Tanzanian researchers to involve insider and outsider perspectives. We sourced data by exploring the perspectives of both TBAs and SBAs. The different methods were chosen in consideration of the environment where participants would feel comfortable, making it easy for them to talk.

### Setting

The study setting was a community in the Korogwe District, which is located in Northeast Tanzania, a rural area of the country. Tanzania comprises 940,000 km^2^. In the past four decades, its population has grown by approximately four times (more than 50,000,000), owing to high birth rates and a decrease in mortality rates [3, 17]. The district of Korogwe is spread over 3756 km^2^ and comprises 132 villages [17]. The main economic activities in the region include agriculture, horticulture (both of which are performed with the natural resources found in the region), and game parks.

In 2012, the Korogwe district had a rural population of 242,038 people and an urban population of 68,308 people [18]. It has one public and two private hospitals, 59 dispensaries, and three health centers. With the support of a local collaborator, the first and second author conducted interviews with TBAs in one of the villages located in a mountain area; the nearest town is 10 km away from the village. They conducted focus group discussion with SBAs in a private room of a district hospital.

### Participants

We used purposeful sampling to identify and collect data from individuals who were information-rich in terms of research purposes. We chose to include both TBAs and SBAs to incorporate the perspectives of both sides. For TBAs, the inclusion criteria were that they must (1) be an active TBA, (2) be able to read and speak Kiswahili, and (3) agree to participate in the study. In our study, SBAs comprised of nurses, midwives, and doctors working in the region. They were invited to participate if they met the inclusion criteria: (1) working in a maternity ward (or working close to this ward), (2) able to speak Kiswahili, and (3) provided consent to participate in the study. For both groups, the exclusion criteria were (1) having never met TBAs, and (2) having no specific perceptions toward this group.

### Data collection

To perform the interviews, the second author asked the village leaders to invite TBAs who worked in their communities to participate in this study. Since we had no previous knowledge of the number of TBAs in the area, we had initially planned to interview all TBAs who eventually appeared in the interview site. Owing to TBAs not being publicly recognized as professionals, we considered that performing group interviews would be a difficult and not very suitable task; hence, we planned individual interviews among this group.

The semi-structured interview guide was created by the first author and later analyzed by the third author to determine if any other questions should be included according to the relevant available literature. The English versions of the interview guides for SBAs and TBAs are attached as supplemental files 1 and 2. After completing the development of the interview questions, the second author translated them from English to Kiswahili. When participants arrived at the interview site, we explained the aims and procedures of the study (including how we would record our conversations) and then asked for their permission to record the interviews. The first author led the interviews in English; the second author acted as an interpreter who translated between English and Kiswahili. The finalized semi-structured interview guide included questions related to TBAs’ activities, recently conducted deliveries, the support they provided to pregnant women, referral cases, their perceptions of TBAs’ roles, their lives aside from the TBA work, and their relationships with healthcare personnel and institutions.

TBA participants came to the village by foot or motorbike. Data collection took place in a classroom of a school in the village. All interviews were conducted with assistance from the second author, acted as an interpreter, who translated between English and Kiswahili. Nonetheless, during the interviews, participants would eventually speak in Kisambaa (the local language); however, the second author (interpreter) understands both languages, so translation was not hindered. The interviews were digitally recorded, transcribed into Kiswahili, and translated into English by the same interpreter. Two authors (the first and fifth authors) reviewed all of the translated data.

For SBAs, we planned focus group discussions (FGDs) so that participants could share their perceptions toward maternal & child health and the roles of TBAs working in the community. The first and second authors acted as the facilitator and the interpreter, respectively. The discussion topics included (1) hospital daily maternal care situations and the corresponding issues and solutions, and (2) the possibility of a collaboration with the TBAs working among pregnant women in rural areas. The FGDs were conducted in Kiswahili, recorded with participants’ consent, and transcribed (in the Kiswahili language). The transcription was later translated from Kiswahili into English by the second author; the fifth author and a Tanzanian assistant checked its accuracy and corrected any issues. Data collection took place in December 2015 for the TBA and August 2016 for the SBAs.

### Data analysis

The qualitative content analysis was guided by the checklist from Elos and Kyngas’ [19] to increase trustworthiness. As they suggest, inductive content analysis was used due to the limited number of previous studies dealing with the phenomenon. The authors put the data in the matrix, which was constructed based on interview aims. For TBA interviews, two authors (the first and third authors) discussed the possible categories and ways to summarize them until they achieved consensus. For the focus group discussions, two authors (the first and fourth authors) discussed the categories until consensus was achieved. Then, similar codes were grouped into sub-categories, and similar sub-categories were grouped into categories. After these two analyses were completed, the first author merged and sorted the final categories according to their similarities and differences. The merged results were shared with the research group (all authors) and received agreement.

### Ethical consideration

Ethical reviews and approvals were obtained from the 1) Institutional Review Board at St. Luke’s International University, Tokyo, Japan (14–040); 2) Director of the Korogwe District Council; 3) National Institute for Medical Research, Tanzania (NIMR/HQ/R.8/Vol.IX/1604); and 4) Tanzania Commission for Science and Technology (COSTECH) (No.2013–273-NA-2013-101).

## Results

### Background information

In total, 15 TBAs came to the site and agreed to participate. They were all female. Their average age was 59.5 years, ranging from 40 to 73. Their education level ranged from the fourth grade to having completed the seventh grade of primary school. In addition to being TBAs, these participants’ occupations were farmer (10), counselor (1), and nurse assistant (1). All participants identified themselves as being Christian. Participants reported becoming TBAs either through training with outside trainers or through learning from older TBAs.

In total, 21 SBAs participated—nine doctors and 12 nurses/midwives. Their age range was 21 to 53, including 13 female and eight male workers. Their work experience ranged from 1 to 30 years.

According to the study purpose, the analyses of the two data sources were combined and sorted into five categories: TBA’s roles, TBA’s knowledge and skills, TBAs’ motivations, issues surrounding TBAs, and TBAs’ needs and ways to progress.

#### TBAs’ roles

TBAs’ descriptions of their own roles included advising women, performing normal deliveries using available resources, and providing referrals. Particularly, TBAs reportedly provided health education on basic hygiene, the importance of going to a hospital, nutrition and exercise for pregnant women, and vaccination. Moreover, they reported being called often when mothers started to have contractions.

*“The work of a traditional birth attendant is to give advice; if the mother is not near [to giving birth], I tell her to go to the hospital.” (TBA-12)*

TBAs said that they would deliver a baby only when they were called to help at the stage of crowning (the baby’s head was about to come out) and in cases where the pregnant women’s family members did not allow them to go to a hospital. Reportedly, they would check the mother and the baby, deliver the baby, provide support to avoid tearing of mothers’ perineum during childbirth, cut the umbilical cord, deliver the placenta, check mothers’ bleeding, and support their breastfeeding. They reported not receiving any materials, such as gloves, plastic sheets, or a blade for cutting umbilical cords, from the official health system, so they use whatever resources are on hand. If a mother has these materials, they use them. If not, the TBAs said they buy the necessary items themselves. For example, they reported using any available clothes to wrap the baby, any available gloves to deliver the baby, plastic sheets as bed covers, a blade to cut and a bandage and stop the bleeding of the umbilical cord, and a brush to wash their hands. Since TBAs help women with the deliveries at home, women’s families tend to play roles in the procedures, such as preparing materials, providing support during delivery, and providing money or food.

*“The family prepares soups and firewood to cook for the mothers.” (TBA-4)*

SBAs’ perceptions of TBAs’ roles were similar: they support pregnant women’s daily lives and provide emergency delivery assistance, health education for community people, and referrals. Nonetheless, they remarked that TBAs could not use oxytocin to stop women’s bleeding after birth nor provide suturing for tears in a woman’s perineum.

*“For example, there is a TBA who, immediately after receiving a pregnant mother, always brings her here [hospital]. Even though she is not yet in labor, she brings her to the hospital and makes sure she is received by care providers. Then, she goes back home, and if [the pregnant mother] does not deliver the same day, the TBA visits her the following day. She follows up.” (SBA: FGD-A)*

#### TBAs’ knowledge and skills

Although participating TBAs and SBAs provided similar descriptions regarding TBAs’ roles, the same could not be said for their statements regarding TBAs’ knowledge and skills. The gaps were clear: TBAs stated that their support for pregnant women did not cause any problems, while SBAs shared their concerns about TBA’s knowledge and skills.

*“No baby that I have delivered has had any bad conditions, but if that happened, I would take the baby to the hospital immediately. There were times when I delivered twin babies and when I delivered two mothers [at the same time], but all were baby boys with good conditions.” (TBA-1)*

*“I assisted a mother whose placenta failed to come out, so I took her to the hospital. I [usually] try to put my hands on the mother’s belly so that the placenta can come out. If it is difficult to remove [the placenta], she should go to the hospital for removal.” (TBA-13)*

One of the SBAs mentioned that TBAs had a kind of strength, as they supported women based on the development and provision of close relationships with the women. However, SBAs were also concerned about TBAs’ lack of formal training and skills and their insufficient knowledge about birth assistance. For example, one SBA talked about a TBA’s inability to monitor labor progress due to their exclusive use of empirical knowledge, as shown herein,

*“What I discovered is that she [the TBA] is using experience. Because she does not know what a partograph is, a TBA acknowledges the time of delivery because she feels that a child is in a certain stage, but [she] doesn’t know the name of the stage. And she doesn’t know if there might be changes or not because she uses experience instead of professional [knowledge].” (SBA:FGD-B)*

Another SBA mentioned that TBAs’ inappropriate practices could endanger babies.

*“She takes a piece of cloth or khanga around her [to wrap the baby’s umbilical cord]. You don’t know how clean it is; it is not sterilized, and, in that case, it is easy for a baby to get a cord infection [infection in the baby’s umbilical cord]. Unfortunately, some [TBAs] do not know how to tighten it well, so you can find some have blood leaking from babies’ umbilical cords. When they arrive at the hospital, you see that the newborn is pale.” (SBA: FGD-B)*

However, as they discussed further, they realized that there was a range of inappropriateness—some TBAs act properly, while others do not provide appropriate care. One SBA acknowledged TBAs’ contributions:

*“The positive side is that they do help [pregnant women], as some areas are far from the facilities, so they help them in their deliveries. As we have seen, some are just doing it because people trust them, but [the issue is] they do not have the skills.” (SBA:FGD-A)*

#### TBAs’ motivations

TBAs stated that what they received as compensation for their work was often only small amounts of money and practical supplies (e.g., soap).

*“Sometimes I am given gifts, like money, [which] I use it to buy other materials, such as gloves.” (TBA-7)*

Rather than receiving compensation, TBAs mentioned that they experienced joy and happiness in their work.

*“I feel good about being a traditional birth attendant; I learn skills, and I also provide close and good service. It is like a first aid service.” (TBA-10)*

*“Many mothers come to me for advice. I feel happy to help my fellow women … . My happiness is when the mother is safe; I become so happy.” (TBA-11)*

Contrastingly, SBAs mentioned that TBAs provide services for their own benefit.

*“You train them [TBAs], but there is an issue in our society regarding the lack of change in behaviors. They remain with [the same] their behaviors, saying, ‘how many [mothers] have we assisted in delivery?’ You [women] should come to us [TBAs]; we will assist you in deliveries here.” (SBA: FGD-B)*

*“They do so [keep delivering babies] because they earn something.” (SBA: FGD-B)*

#### Issues surrounding TBAs

TBAs stated that the issues surrounding their activities amounted to a lack of resources and unfavorable relationships with the hospital. Especially, they repeatedly mentioned that SBAs presented negative attitudes toward them.

*“There is no cooperation with health personnel at all. When we go to the hospital, they chase us out.” (TBA-9)*

Another TBA talked about the lack of inclusion in training.

*“Our fellow colleagues from the hospital do not invite us to their activities. We want to learn because, when the mother is sick, we don’t know any of the signs, even if they are written on the antenatal cards.” (TBA-6)*

Another TBA talked about the lack of rewards when they referred mothers to a hospital.

*“When I transfer [a mother] to the hospital, she does not give me anything. She gives me money or gifts only if I can conduct the delivery at home.” (TBA-6)*

Conversely, SBAs shared their concerns about TBAs’ activities, which are performed without proper knowledge, and how their interventions may affect the professional work of SBAs.

*“I think many professionals have experienced interference [in their jobs] as she [a TBA] tells women not to go to health facilities early because they [healthcare providers in a hospital] will provide a Cesarean section. She tells women to come to the TBAs [not the hospital]. These are the negative attitudes.” (SBA: FGD-B)*

Another SBA mentioned that pregnant women often rely on the elder TBAs’ health knowledge, whereas TBAs often delay referral to a hospital owing to their overall lack of knowledge about healthcare and childbirth.

*“Some [TBAs] are doing it [delivery of babies] because they may be seen as elders, so young women are sent to them for delivery; but they know nothing, so they stay with women until they demonstrate complications, resulting in a delayed referral.” (SBA: FGD-A)*

#### TBAs’ needs and ways to progress

TBAs mentioned that they need further training for their normal delivery skills: They would like to receive more training on cervix dilation, delivery timing, injections to stop bleeding, and blades for cutting umbilical cords. They also mentioned that novel and aspiring TBAs need training as many of the participating TBAs are getting older.

*“We, as traditional birth attendants, should cooperate. I want to teach young traditional birth attendants, so that they may know what we do and help mothers together.” (TBA-8)*

In this regard, SBAs concurred with TBAs once more. To solve their concerns, an SBA stated that they need to provide TBAs with continuous training and to check their abilities regularly. Additionally, SBAs discussed the need for the promotion of collaboration and communication with TBAs, which could be provided by holding regular meetings to disseminate updated information.

*“It is important to meet them and give them continuous education because they have experience; so, by giving them continuous education, they will be aware of current updates, like infection prevention [methods].” (SBA: FGD-B)*

TBAs stated that they needed more materials, including gloves, plastic bed covers, and fetoscopes, to perform their work. Furthermore, the SBAs are concerned that the use of regular (not disinfected) tools could cause infections in mothers, babies, and even the TBAs themselves. Hence, SBAs mentioned the need for providing TBAs with medical supplies, including medical scissors, clips to clamp the baby’s umbilical cord, and gloves.

*“Sometimes they use banana threads. They need tools like these surgical blades if they [have to] cut and clamp the cord, and the gloves because they sometimes receive the babies directly, without any protection.” (SBA: FGD-A)*

Moreover, SBAs provided a specific statement that was not likewise provided by the TBAs. They said that TBAs needed to receive support because they spend a lot of time on TBA activities, which they could use for farming and other money-making activities otherwise.

*“They also need support because they are using their time. Some eventually stay the whole night with a patient because of the distance from [the house to] the facility. They [TBAs] need to receive something to support them because they have stopped doing their other tasks to handle a patient. So, if it is possible [to provide support], they will feel respected.” (SBA: FGD-A)*

The final discussions in the focus group discussion regarded the importance of collaboration between these two groups.

*“If TBAs started collaborations with clinic providers, the nearby facilities may perform well because clinic providers could meet TBAs and continue to train and educate them on delivery to receive a pregnant mother [at the proper time]. TBAs are good! Also, TBAs are good because they are the ones who stay with their [community] people in the peripheral areas.” (SBA: FGD-A)*

*“I think the main thing is to expand services to the people. For example, by having health facilities provide TBAs with working tools, we will reduce maternal mortality.” (SBA: FGD-A)*

## Discussion

This study illustrates the continuing contributions of TBAs to maternal-child health in the community in Tanzania, despite their role not being recognized in the formal healthcare system. Additionally, our study highlights the wide range of opinions and perceptions of TBAs and SBAs (who are directly and indirectly affected by TBAs’ work) regarding TBAs’ roles. At the time of data collection, it seemed like there was a gap in the communication between the two groups; nonetheless, both groups seemed willing to collaborate and interact in order to ensure mothers and babies are healthy and to preserve lives.

Until 1999, TBAs did receive training on the outskirts of the village [20]. Our results showed that, regarding TBAs’ roles, TBAs’ and SBAs’ descriptions did not differ. However, they did differ regarding TBAs’ knowledge and skill levels. TBAs claimed their compliance with the training they had received before, and SBAs repeatedly mentioned TBAs’ lack of training caused interference in SBAs’ practice. However, possibly owing to the international and national policy changes that ended TBA training and support, TBAs may have started to experience a greater lack of resources and the loss of the ability to update their knowledge. They also encounter barriers to collaboration with SBAs in the formal healthcare system. Nonetheless, given that the need for collaboration was not denied by any of the participating groups, the current issue may relate more to the “Hows” that will allow them to achieve such collaborations. We believe that these changes could assist in the promotion of universal healthcare access by reaching even those who are at the most remote places where resources are scarce, and development is direly needed [21].

Regarding possible practical collaborations between SBAs and TBAs, we acknowledge that these will vary according to the context. The most common approach involves providing TBAs with incentives for bringing pregnant women to health facilities. In Somaliland, a study provided TBAs with a 3-day training, a 1-year follow-up training, and temporary incentives when they brought pregnant women to a healthcare facility [12]. In this cited study, 75 TBAs participated, and the training improved not only their skills but also their knowledge and scope of practice, improving their ability to identify danger signs more precisely. Additionally, they kept bringing pregnant women to the healthcare facility even after the temporary incentives were no longer provided [12]. A similar study conducted in Kenya also resulted in an increase in the number of times TBAs brought pregnant women to health facilities when incentives were provided [11].

Additionally, we would like to highlight other inclusive approaches that were used in previous studies. In East Timor, to improve maternal-child health in the communities, a volunteer leader was chosen for a community. This leader received training on maternal-child health activities and, afterward, was asked to provide health education, home visits to pregnant women, and to accompany pregnant women to a health facility [22]. In the cited study, TBAs were included as volunteer leaders, and, although they did not receive salaries, they received incentives, continuous training, and material supplies. The results showed that the leaders acted as linking points between the communities and health facilities, hence enabling facility-community collaboration [22]. In Uganda, TBAs were included in the healthcare team [23]. Although it was an exceptional approach, they acknowledged that it occurred because TBAs had been contributing to the community for a very long time. Once they were recognized as team members, TBAs started to feel willing to be more compliant with SBAs; moreover, the strong trust that existed between community members and TBAs was beneficial for improving maternal healthcare services [23].

Given that both groups in our study reported the need for TBAs to receive training and the need for collaboration between SBAs and TBAs, we believe that the next step relates to planning programs that bridge the current gaps in perception described in the findings. In Miller and Smith’s review, it was reported that traditional communities face resistance to changes, and negative attitudes were observed between TBAs and SBAs [24]. The authors pointed out that a facilitating factor was stakeholder involvement (e.g., decision-makers in the households and community/religious leaders) [24]. Given that this study found similar negative attitudes between TBAs and SBAs, we believe that a program aimed at bridging the gaps between these groups should be conducted in order to make efforts to involve community leadership. Regarding such involvement, the cited authors suggested that these leaders should be provided with formalized roles and responsibilities, that TBAs should be remunerated, and that SBAs and TBAs should share their knowledge with one another [24].

Regarding the implementation of such recommendations, a previous study has shown that, to realistically try to implement foreign knowledge in traditional communities, one needs to understand the existing local knowledge and reference the social, political, and cultural context of these communities [25]. Using traditional practices in Peru from the late 18th to early 19th centuries as examples, Warren [25] pointed out that, although a French midwife already emphasized external “correct” knowledge and practice at that time, local women, even those highly educated, still sought care from and relied on traditional midwives [25]. A literature review on Asian traditional beliefs and practices also described that cultural beliefs can influence women’s use of formal maternal healthcare services, and that women’s fear of unnecessary medical interventions was a barrier to institutional births [26]. In a review of childbirth and postnatal care in rural Africa, the authors found that the influencing factors of people’s preferences regarding formal or traditional healthcare were related to perceived attitudes toward healthcare providers, accessibility to maternal care, and respect for cultural and religious norms [27]. The study also demonstrated that women across rural Africa prefer to receive childbirth care from caring, considerate, and sympathetic providers, and that this population group sees cruel, insensitive, and degrading attendants as increasing negative maternal experiences and outcomes. A final remark from the cited study highlighted that the trust rural African women have in TBAs influenced the type of healthcare service they sought [27].

Given these considerations, the World Health Organization published guidelines to promote a positive pregnancy experience based on the fact that women repeatedly report disrespect and abuse during childbirth in health facility [28, 29]. The accumulated evidence from these systematic review papers showed how increased workload of SBAs due to policy change in 90’s led to the inability to provide a birth environment where the needs of birthing women could be met. The new guidelines include maintaining women’s physical and sociocultural normality, maintaining a healthy pregnancy for both mother and baby (i.e. preventing and treating risk factors, illness and death), providing an effective transition to positive labor and birth, and assisting women in achieving positive motherhood (i.e. maternal self-esteem, competence, and autonomy) [30]. This shift from focusing on survival to focusing on helping mothers thrive through the enhancement of the mother-practitioner trust relationship shows the expanded potential roles of TBAs. These roles may be included in the “maintaining women’s physical and sociocultural normality” category, as TBAs can serve as their local knowledge providers, hence contributing to women’s sense of normality and health.

Walsh et al. [31] discussed types of favored and effective traditional leadership using examples from Malawi. When fear and coercion were justified, community leaders often used them to ensure that women delivered at health facilities. Hence, the authors concluded that, although a leadership empowerment model is desirable, it may be unfeasible to implement in highly hierarchical cultures. Conversely, a recent study among Maasai tribes in Tanzania described the need to rebuild trust among SBAs, TBAs, and the community [32]. That study emphasized that increased sensitivity to women’s cultural preferences could close the gap in the trust between these stakeholders. Therefore, it would be appropriate to try a leadership empowerment model using TBAs’ local knowledge and trust to assist women in the community regarding healthcare.

The next step would be to have a meeting of both sides and discuss what is really needed in the community. The discussion should include how they could work together so that both sides could be cooperative at the time of referral and at the time of maternal education. This can be implemented as an action research so that changes in maternal healthcare service and improvement in maternal health would occur.

Although this study has its strength in design using triangulation, some limitations remain. We did not utilize the member-checking procedure because returning to TBAs for confirmation of analysis was not feasible due to language barriers and a lack of communication methods. We did not seek responses from TBAs based on the SBAs’ opinions. Hence, we do not have information to report on the TBAs’ response to the SBAs’ concerns. However, the first author has been conducting research in Tanzania for more than 10 years and understands the Kiswahili language. Likewise, three of the authors are Tanzanian midwifery researchers who provide local perspectives and cultural understanding. Nevertheless, further research is needed to inform the development of a program that bridges the gaps between TBAs and SBAs in Tanzania.

## Conclusion

TBAs and SBAs described different perceptions on TBAs’ knowledge and skills. Although TBAs’ inclusion into the formal healthcare system has yet to be established, both groups acknowledged that TBAs should be provided with training and inclusion. TBAs were shown to have the potential to contribute to SBAs’ work through their local knowledge. Specifically, they may be more prepared to support women’s cultural preferences. Such preparedness added to SBAs’ provision of compassionate care, could allow mothers in rural Tanzania to build their trust toward SBAs and to have more positive birth experiences. We believe that such practical modifications could help in achieving the goal of providing universal access to maternal healthcare in the country.

## Supplementary Information

**Additional file 1.** Interview Guide for Skilled Birth Attendants

**Additional file 2.** Interview Guide for Traditional Birth Attendants

## Data Availability

The datasets used and analyzed during the current study are available from the corresponding author upon reasonable request.

## Abbreviations

TBAs: Traditional Birth Attendants
SBAs: Skilled Birth Attendants
FGD: Focus Group Discussion
MMR: Maternal Mortality Ratio

## References

[1] United Nations (2020). Sustainable Development Goals.

[2] Ministry of Health, Community Development, Gender, Elderly and Children (MoHCDGEC) [Tanzania Mainland], Ministry of Health (MoH) [Zanzibar], National Bureau of Statistics (NBS), Office of the Chief Government Statistician (OCGS), and ICF (2016). Tanzania demographic and health survey and malaria indicator survey (TDHS-MIS) 2015–16.

[3] Kwesigabo G, Mwangu MA, Kakoko DC, Warriner I, Mkony CA, Killewo J, Macfarlane SB, Kaaya EE, Freeman P (2012). Tanzania’s health system and workforce crisis. J Public Health Policy.

[4] Sibley LM, Sipe TA (2006). Transition to skilled birth attendance: is there a future role for trained traditional birth attendants?. J Health Popul Nutr.

[5] Wilson A, Gallos ID, Plana N, Lissauer D, Khan KS, MacArthur C, et al. Effectiveness of strategies incorporating training and support of traditional birth attendants on perinatal and maternal mortality: meta-analysis. BMJ. 2011. 10.1136/bmj.d7102. 10.1136/bmj.d7102 PMC3228291 22134967

[6] Sibley LM, Sipe TA, Barry D. Traditional birth attendant training for improving health behaviours and pregnancy outcomes. Cochrane Database Syst Rev. 2012. 10.1002/14651858.CD005460.pub3. 10.1002/14651858.CD005460.pub3 PMC4158424 22895949

[7] World Health Organization, United Nations Population Fund & United Nations Children’s Fund (UNICEF) (1992). Traditional birth attendants: A joint WHO/UNFPA/UNICEF statement.

[8] United Nations Population Fund (1997). Report of the executive director for 1996: Evaluation activities.

[9] Ministry of Health, Community Development, Gender, Elderly and Children (MoHCDGEC) (2016). The national road map strategic plan to improve reproductive, maternal, newborn, child & adolescent health in Tanzania (2016–2020): ONE PLAN II.

[10] Mahiti GR, Kiwara AD, Mbekenga CK, Hurtig AK, Goicolea I. “We have been working overnight without sleeping”: traditional birth attendants’ practices and perceptions of post-partum care services in rural Tanzania. BMC Pregnancy Childbirth. 2015. 10.1186/s12884-015-0445-z. 10.1186/s12884-015-0445-z PMC4324777 25643622

[11] Kitui JE, Dutton V, Bester D, Ndirangu R, Wangai S, Ngugi S. Traditional birth attendant reorientation and motherpacks incentive’s effect on health facility delivery uptake in Narok County, Kenya: an impact analysis. BMC Pregnancy Childbirth. 2017. 10.1186/s12884-017-1307-7. 10.1186/s12884-017-1307-7 PMC5399849 28431565

[12] Pyone T, Adaji S, Madaj B, Woldetsadik T, van den Broek N. Changing the role of the traditional birth attendant in Somaliland. Int J Gynaecol Obstet. 2014. 10.1016/j.ijgo.2014.04.009. 10.1016/j.ijgo.2014.04.009 24938771

[13] Orya E, Adaji S, Pyone T, Wurie H, van den Broek N, Theobald S. Strengthening close to community provision of maternal health services in fragile settings: an exploration of the changing roles of TBAs in Sierra Leone and Somaliland. BMC Health Serv Res. 2017. 10.1186/s12913-017-2400-3. 10.1186/s12913-017-2400-3 PMC5498892 28679383

[14] Jiang H, Qian X, Chen L, Li J, Escobar E, Story M, et al. Towards universal access to skilled birth attendance: the process of transforming the role of traditional birth attendants in rural China. BMC Pregnancy Childbirth. 2016. 10.1186/s12884-016-0854-7. 10.1186/s12884-016-0854-7 PMC4800763 27000104

[15] Talukder S, Farhana D, Vitta B, Greiner T. In a rural area of Bangladesh, traditional birth attendant training improved early infant feeding practices: a pragmatic cluster randomized trial. Matern Child Nutr. 2017. 10.1111/mcn.12237. 10.1111/mcn.12237 PMC6865915 26775711

[16] Carter N, Bryant-Lukosius D, DiCenso A, Blythe J, Neville AJ. The use of triangulation in qualitative research. Oncol Nurs Forum. 2014. 10.1188/14.ONF.545-547. 10.1188/14.ONF.545-547 25158659

[17] The Planning Commission Dar es Salaam & Regional Commissioner’s Office (1997). Tanga Region Socio-Economic Profile.

[18] National Bureau of Statistics, Ministry of Finance, Dar es Salaam, and Office of Chief Government Statistician, President’s Office, Finance, Economy and Development Planning (2012). Population and housing census.

[19] Elo S, Kyngäs H. Qualitative content analysis: a focus on trustworthiness. SAGE Open. 2014. 10.1177/2158244014522633.

[20] JICA (1999). Report of the outcome evaluation of the maternal child health project in the United Republic of Tanzania.

[21] United Nations Development Programme and United Nations Capital Development Fund (2016). Getting to the last mile in least developed countries.

[22] Ribeiro SD. Traditional birth attendance (TBA) in a health system: what are the roles, benefits and challenges: a case study of incorporated TBA in Timor-Leste. Asia Pac Fam Med. 2014. 10.1186/s12930-014-0012-1. 10.1186/s12930-014-0012-1 PMC4251863 25469105

[23] Rudrum S. Traditional birth attendants in rural northern Uganda: policy, practice, and ethics. Health Care Women Int. 2016. 10.1080/07399332.2015.1020539. 10.1080/07399332.2015.1020539 25719535

[24] Miller T, Smith H. Establishing partnership with traditional birth attendants for improved maternal and newborn health: a review of factors influencing implementation. BMC Pregnancy Childbirth. 2017. 10.1186/s12884-017-1534-y. 10.1186/s12884-017-1534-y PMC5649078 29052533

[25] Warren A. Between the foreign and the local: French midwifery, traditional practitioners, and vernacular medical knowledge about childbirth in Lima, Peru. Hist Cienc Saude Manguinhos. 2015. 10.1590/S0104-59702015000100011. 10.1590/S0104-59702015000100011 25742106

[26] Withers M, Kharazmi N, Lim E. Traditional beliefs and practices in pregnancy, childbirth and postpartum: a review of the evidence from Asian countries. Midwifery. 2018. 10.1016/j.midw.2017.10.019. 10.1016/j.midw.2017.10.019 29132060

[27] Fantaye AW, Gunawardena N, Yaya S. Preferences for formal and traditional sources of childbirth and postnatal care among women in rural Africa: a systematic review. PLoS One. 2019. 10.1371/journal.pone.0222110. 10.1371/journal.pone.0222110 PMC6760778 31553722

[28] Bohren MA, Berger BO, Munthe-Kaas H, Tunçalp Ö. Perceptions and experiences of labor companionship: a qualitative evidence synthesis. Cochrane Database Syst Rev. 2019. 10.1002/14651858.CD012449.pub2. 10.1002/14651858.CD012449.pub2 PMC6422112 30883666

[29] Bradley S, McCourt C, Rayment J, Parmar D (2016). Disrespectful intrapartum care during facility-based delivery in sub-Saharan Africa: a qualitative systematic review and thematic synthesis of women's perceptions and experiences. Soc Sci Med.

[30] World Health Organization (2016). WHO recommendations on antenatal care for a positive pregnancy experience.

[31] Walsh A, Matthews A, Manda-Taylor L, Brugha R, Mwale D, Phiri T, et al. The role of the traditional leader in implementing maternal, newborn and child health policy in Malawi. Health Policy Plan. 2018. 10.1093/heapol/czy059. 10.1093/heapol/czy059 30084938

[32] Mosley PD, Saruni K, Lenga B. Factors influencing adoption of facility-assisted delivery - a qualitative study of women and other stakeholders in a Maasai community in Ngorongoro District, Tanzania. BMC Pregnancy Childbirth. 2020. 10.1186/s12884-020-2728-2. 10.1186/s12884-020-2728-2 PMC7014728 32050919

