# Supplementary material for: Perceived differences on the role of traditional birth attendants in rural Tanzania: a qualitative study
Source: BMC Pregnancy Childbirth. 2021 Feb 15;21:137. doi: 10.1186/s12884-021-03611-0 (PMC7885621; doi:10.1186/s12884-021-03611-0)
Supplement: Supplementary file 1 — Additional file 1. Interview Guide for Skilled Birth Attendants [file 12884_2021_3611_MOESM1_ESM.docx]

Interview Guide for Skilled Birth Attendants

Purpose

1. To identify the situations of maternity care in the hospital and discuss the issues and solutions.

2. To discuss if they can collaborate with TBAs who work for pregnant women in rural areas.

<Background information>

1. Age
2. Education
3. Professional category
4. Years at work
5. Regular work place

<Antenatal clinic>

1. What do you examine pregnant women for at the antenatal clinic? (e.g., weight, blood pressure, etc.)

2. What do you tell expectant mothers?

3. Are there any other things that you think are important to tell mothers?

<Deliveries>

1. Please tell me about the most recent delivery that is most memorable for you.
2. Please also tell me about the woman (age, education, parity, ethnic group, religion).
3. How did the woman arrive at the hospital?
4. How did the delivery progress?
5. What did you do to care for this woman?
6. What kind of materials do you use for delivery? How did you get them?
7. Were there any family members? What did the family do for this woman?
8. How did the delivery finish? Were both mother and baby safe?

<Abnormal, referral cases>

1. Have you ever experienced abnormal cases or problems with mothers and babies? If so, what kinds of problems? How did you deal with the cases?
2. In the end, what happened to the mother and the baby?

<Issues in the ward>

1. Please tell me about the issues or difficulties in your ward (too many patients, too many complications, lack of materials, etc.).
2. What is the reason to cause the issues or difficulties?
3. How do you think you can solve the issues or difficulties?
4. What do you think about maternal mortality in this area?
5. How do you think we can reduce maternal mortality?

<Administration>

1. Please tell me about your work. What do you do everyday?

2. Are you familiar with the issues of maternal mortality? If so what do you think about them?

3. What are the issues related to maternal care? (quality of care, referral system, knowledge of community people, etc.)

4. What are the policies and related activities in maternal child health?

5. Does maternal child health have enough budget?

<Perception toward TBAs>

1. Do you know that TBAs exist around this area?
2. How do you think about TBAs? (positive or negative, etc.)
3. How did you receive a woman when a TBA referred her?
4. Have you ever experienced anything good or bad with TBAs?
5. How do you think you can work better with TBAs?
6. Are you willing to attend if we set a meeting with TBAs?
7. What do you think they need to work better as a TBA? (materials, support, training, etc.)
