# Supplementary material for: Perceived differences on the role of traditional birth attendants in rural Tanzania: a qualitative study
Source: BMC Pregnancy Childbirth. 2021 Feb 15;21:137. doi: 10.1186/s12884-021-03611-0 (PMC7885621; doi:10.1186/s12884-021-03611-0)
Supplement: Supplementary file 2 — Additional file 2. Interview Guide for Traditional Birth Attendants [file 12884_2021_3611_MOESM2_ESM.docx]

Interview Guide for Traditional Birth Attendants

Purpose

To identify how TBAs perceive their own activities and how they can connect with the formal health system.

<Background information>

1. Age
2. Family
3. Education
4. Other job
5. Ethnic group
6. Religion

<Activities of TBA>

1. What are your activities as a TBA?
2. How many deliveries have you conducted before?
3. Are you currently continuing the activities? If so, how many deliveries per month?
4. How did you become a TBA? Were you asked to become one or did you want to become one?
5. Have you ever gotten any training? If so, what kind of training and when?

<Deliveries>

1. Please tell me about the most recent delivery.
2. Please also tell me about the woman (age, education, parity, ethnic group, religion).
3. How did the woman contact you?
4. How did this woman reach you? Or did you go to her house?
5. How did the delivery progress?
6. What did you do to care for this woman?
7. What kind of materials do you use for delivery? How did you get them?
8. What did the family do to care for this woman?
9. How did the delivery finish? Were both mother and baby safe?
10. Did you receive anything after the delivery? Do you usually receive anything? Like what?

<Pregnancy>

1. Did you take care of the woman when she was pregnant? If so, how did you care for her?
2. Did she also go to an antenatal clinic?
3. Do you usually take care of pregnant women? If so, how do you take care of pregnant women?

<Abnormal, referral cases>

1. Have you ever experienced abnormal cases or problems with mothers and babies? If so, what kinds of problems? How did you deal with the cases?
2. In the end, what happened to the mother and the baby?

<Perception as TBA>

1. How do you feel about being a TBA? (satisfaction, responsibility, nervousness, etc.)
2. What do you think you need to work as a TBA? (materials, support, training, etc.)

<Usual life and communication being a TBA>

1. How do you spend your day when you are not working as a TBA?
2. Do you have any communication with other TBAs? If so, what do you do?
3. Do you train or teach young TBAs?

<Hospital>

1. What do you think about health centers or hospitals?
2. What do you think about nurses or doctors at hospitals?
3. Do you have any connection with the formal health system? If so, what kind of connection?
4. How do you hope to connect with them from now?
